# Grower decision-making factors in adoption of specialty cultivars: A case study of potatoes in the San Luis Valley

**DOI:** 10.1371/journal.pone.0270636

**Published:** 2022-06-30

**Authors:** Sahar B. Toulabi, Becca Jablonski, David G. Holm, Michael S. Carolan, Adam L. Heuberger

**Affiliations:** 1 Department of Horticulture and Landscape Architecture, Colorado State University, Fort Collins, Colorado, United States of America; 2 Department of Agriculture and Resource Economics, Colorado State University, Fort Collins, Colorado, United States of America; 3 Department of Sociology, Colorado State University, Fort Collins, Colorado, United States of America; 4 Department of Soil and Crop Sciences, Colorado State University, Fort Collins, Colorado, United States of America; Universidad Nacional Autonoma de Nicaragua Leon, NICARAGUA

## Abstract

Potatoes are the most consumed vegetable worldwide and play an important role in the U.S. economy. Growers make critical decisions each year in choosing which cultivar to grow, based on factors such as yield, resilience to the growing environment, and utility in the food industry. Current research supports the finding that less-common specialty cultivars (SCs) have benefits for human health. However, growers have been slow to adopt SCs into mainstream operations. Here, we identify major factors in the decision-making process that determine whether a population of growers in the San Luis Valley, Colorado, a major potato-growing region, adopt SC potatoes. We used a combination of ethnographic techniques and quantitative methods to examine drivers of adoption. The data demonstrate grower perceptions within potato farming and the complexity of interacting factors in decision-making. An integration of the Theory of Planned Behavior, Rational Expectation Hypothesis, and Diffusion of Innovation models identifies economic and social factors that influence grower decision-making. Growers that were more aware of specialty cultivar innovation and associated consumer demand were more open to SCs adoption. Other influencing factors include a grower’s experience selling a SC in the previous year and access to diverse markets. Based on these data, we developed a new model to explain grower decision-making processes in adopting SCs. The model demonstrates that one current barrier to adoption is access to buyers, including warehouses, retailers, and households. Taken together, this research demonstrates how rational expectations stem from economic outcomes, knowledge, and experience in the potato industry. These results are important in helping to consider opportunities for growers to access new, higher value markets, while also improving consumer access to nutritious cultivars.

## Introduction

Potatoes are the most consumed vegetable crop in the United States (U.S.) and represent an important segment of the U.S. agricultural economy. In 2020, the U.S. fresh and processed potato market was valued at U.S.$3.90 B [1], and the U.S. had $1.9 B in exports as the fifth largest producer in the global market after China, India, Russia, and Ukraine[2]. The U.S. state of Colorado is the fifth largest potato producer by sales, and is the second largest shipping state, with sales of $209 M in 2020 [3]. In Colorado, most potato production (94%) occurs in the San Luis Valley (SLV) [4]. The SLV is geographically optimal for potato production due to its high elevation, nearly complete enclosure by surrounding mountains, and unique high, sandy loam soils. Potato production makes up a significant portion of the gross domestic product for the region. And, the region is economically challenged, including reporting higher rates of poverty than either CO or the U.S. [5]

Despite its place as the top consumed vegetable, consumption of fresh potatoes has been in decline, falling by about 20% during the 1970’s, before stabilizing during the 1980s and 1990s and trending lower again since 2000. These long-term trends reflect changes in the market as well as dietary shifts, including greater availability of processed potatoes (especially frozen) that supplant consumption of fresh potatoes, and growing interest in low-carbohydrate diets. At the same time, there is growing consumer interest in and demand for fruits and vegetables with novel health attributes (e.g., [6, 7]). Salehi (2021), for example, finds that there is growth in demand for foods that improve diet, including those rich in compounds with antioxidant activity and biological properties.

In agriculture, research and innovation function to improve food crops for yield, disease resistance, and resilience in order to grow in challenging climates. Plant breeding, the process of crossing plants to introduce new combinations of genetics in the food system, plays an important role in producing new cultivars (sometimes referred to as a crop “variety”). Most of the cultivars that are released each year are minor variants of already-established genetics, usually with small changes, such as better yield, disease resistance, or processing traits. However, plant-breeding operations often concurrently breed for “specialty” cultivars (SCs). These cultivars often have lower yields and different traits than cultivars typically grown by the industry (e.g., size, shape, color, etc.), but they excel at value-added traits [8–10]. In potatoes, one novel value-added trait is related to human health. Most traditional potato cultivars lack color, but some SC potatoes are rich in anthocyanins (i.e., purple or red flesh cultivars), carotenoids (i.e., dark orange flesh cultivars), and other phytochemicals that have demonstrated preventative effects on cardiovascular disease and metabolic syndrome [11–14]. Each year, growers must decide which cultivars to grow. This decision may be considered vital to public health given that potatoes remain the most consumed vegetable [15–19].

Adopting a new approach by either using a new technology or adopting a new crop brings risk to the system and is therefore associated with complex psychological and economic factors. Previously identified factors that influence risk and adoption include sociodemographic (e.g., grower age, education level) [20–23], socioeconomic (e.g., size and diversity of a grower’s operation, access to resources) [24, 25], and circumstantial factors (e.g., social networks) [24, 26]. As such, it is necessary to develop a multifactorial model to explain adoption in each agronomic system.

Here, we hypothesize that socioeconomic, social, and cognitive factors influence the decision-making process for growers in adopting SCs. This research investigates grower willingness to adopt SCs within the SLV. Importantly, the SLV is also the location of the Colorado State University Potato Breeding and Selection Program, which acts as the main source of potato research and innovation in the area. The program has released 34 cultivars since 1975, and many of the SCs have unique nutritional chemistries and improved human health traits. Therefore, we conducted this analysis to evaluate the barriers to growers adopting specialties generated by this center of innovation.

## Materials and methods

### Theoretical framework

We integrate three established models of innovation to understand the decision-making process for growers to adopt new potato cultivars. We analyzed components of each model independently and then combined them in a final model. Here, we present a theoretical framework and central definitions of the model.

#### Concept of innovation

Innovation is a new object or idea applied to initiating or improving a product, process, or service [27]. Early innovation models were linear or “science pull” models in which the idea and innovation started from scientific and research organizations, flowed to technology (applied science), and then to markets [28, 29]. Today, the innovation model works by coupling the interactions between science and technology and the marketplace, along with feedback loops. In these more complicated models, ideas and needs are generated both inside and outside of research firms, with several “go” or “kill” decision points [29, 30]. Individuals and organizations involved in the development, manufacturing, sale, and use of innovations must interact at these stages/gates because missing these interactions in the process will cause innovations to fail to perform [30].

#### Concept of adoption and related models

**Fig 1:**
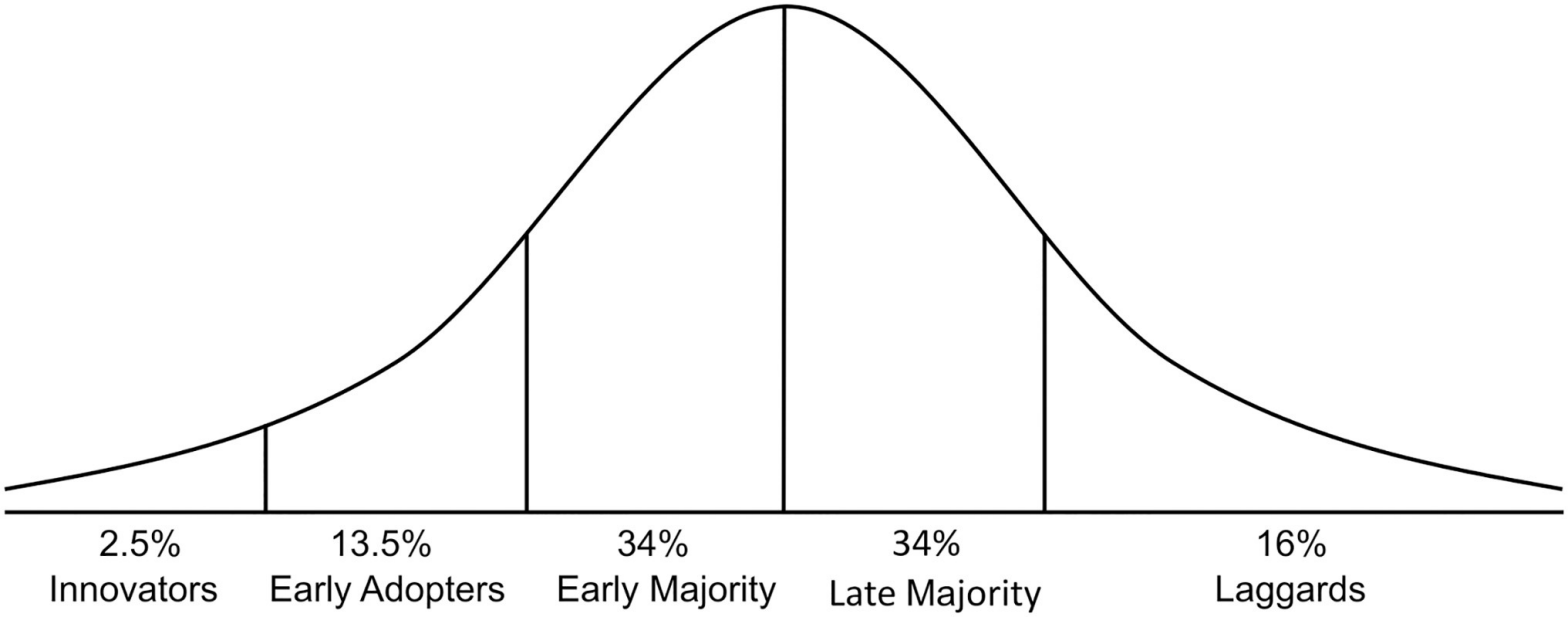
Diffusion of innovation model from Rogers (1962).

An adopter is someone who decides to invest their resources (e.g., time, money, operation) in an innovation (e.g., technology or new idea). Many different theories and models in social and behavioral sciences explain the innovation adoption processes of consumers and producers. Rogers’ Diffusion of Innovation (DOI) model, the most popular innovation model, explains that adoption and innovation do not happen simultaneously. A new idea (innovation) gains momentum over time and diffuses (or spreads) through specific populations or social systems, as individuals have different approaches to and readiness for it. The DOI model divides people into five classes based on their adoption willingness and readiness and explains changes in the flow and rate of adoption based on several factors, including communication channel, social system, and attribute of innovation (e.g., comparability and complexity) (Fig 1) [31, 32].

Adoption of an innovation usually brings financial risk to an organization or farm, which leads to uncertainty about whether to invest in the innovation. Some economic models have tried to explain the decision to adopt. For example, the Rational Expectations Hypothesis (REH) suggests that a grower has a rational self-interest in maximizing economic returns [33]. Accordingly, expectations of future profit will play an important role in the grower’s decision to adopt. Decision-makers thus use a set of information (belief, knowledge, and experience) to evaluate and predict an outcome to decide whether they will invest their resources [34–36]. Another theory, the Theory of Planned Behavior (TPB), has been applied to predict human behavior. It explains that the intention to carry out a certain behavior is determined by three central psychological predictors: attitude, subjective norms, and perceived behavioral control [37]. The TPB has been applied extensively to explain and predict the adoption of an innovation and decision-making process in sustainable agricultural and clean technology [38–41], land management practices [42, 43], natural resource management [44], water management [45], animal welfare practices [46], organic farming [47], and precision agriculture [48].

#### Model component 1: Attitudes and behavioral beliefs

A key variable and fundamental build block of behavioral change is a person’s attitude—a person’s positive or negative feelings and evaluation of a perceived outcome. Behavioral beliefs (BB) are beliefs about the likelihood of a certain outcome (i) of a behavior (b) and the evaluation of these outcomes (e) $\left(BB=\sum_{i=1}^{n}biei\right)$ [43, 49, 50]. Several studies report that attitude is a significant predictor of intention to adopt, and individual attitudes are found to differ across adopters and non-adopters [45, 51, 52], including grower’s knowledge and experience with innovation, their age, and their education [44, 53–57].

#### Model component 2: Subjective norms

Subjective norms (SN) encompass individual perceptions and beliefs about the views of others (called reference group j). In a social system or network members tend to consider the perspectives of other members and related groups when forming their own attitudes towards a given behavior [24, 44, 51, 58, 59]. Subjective norms are determined by Normative Beliefs (NB) (nj X mj), which are the normative expectations of a reference group (nj), and the motivation (m) to comply with the opinion of these referents to carry out the behavior $\left(SN=\sum_{j=1}^{n}njmj\right)$ [43, 50]. Several theoretical models have been developed to explain determinants of social influence on individual behavior (e.g., the theory of normative social behavior, the structural theory of social influence [60, 61].

A grower’s network has been found to significantly affect their intention to adopt a new practice [24, 44, 62–65]. In addition, factors such as education level, age, economic status, network size, participation in social activities, and interactions with others have been found to impact normative beliefs [24, 66].

#### Model component 3: Perceived behavioral control

Perceived Control (PC) is an individual’s perception of their capacity to conduct successful performance and behavior, including the ease or difficulty implementing a particular behavior or the extent to which they feel in control of the decision-making process [44, 67, 68]. Belief about the presence of different factors (k) can facilitate or inhibit the execution of certain behavior (c). The control belief also results from the perceived power of the (k_th_) factor to facilitate or inhibit the behavior (p) $\left(PC=\sum_{k=1}^{n}ckpk\right)$ [43, 50, 68]. A grower’s possession of knowledge and training to use an innovation are factors found to influence their belief in their ability to control and manage risk [45, 69]. Growers integrate and evaluate their ability to control the financial outcomes, successes, and risks of innovation [44].

#### Development of an integrated framework

**Fig 2:**
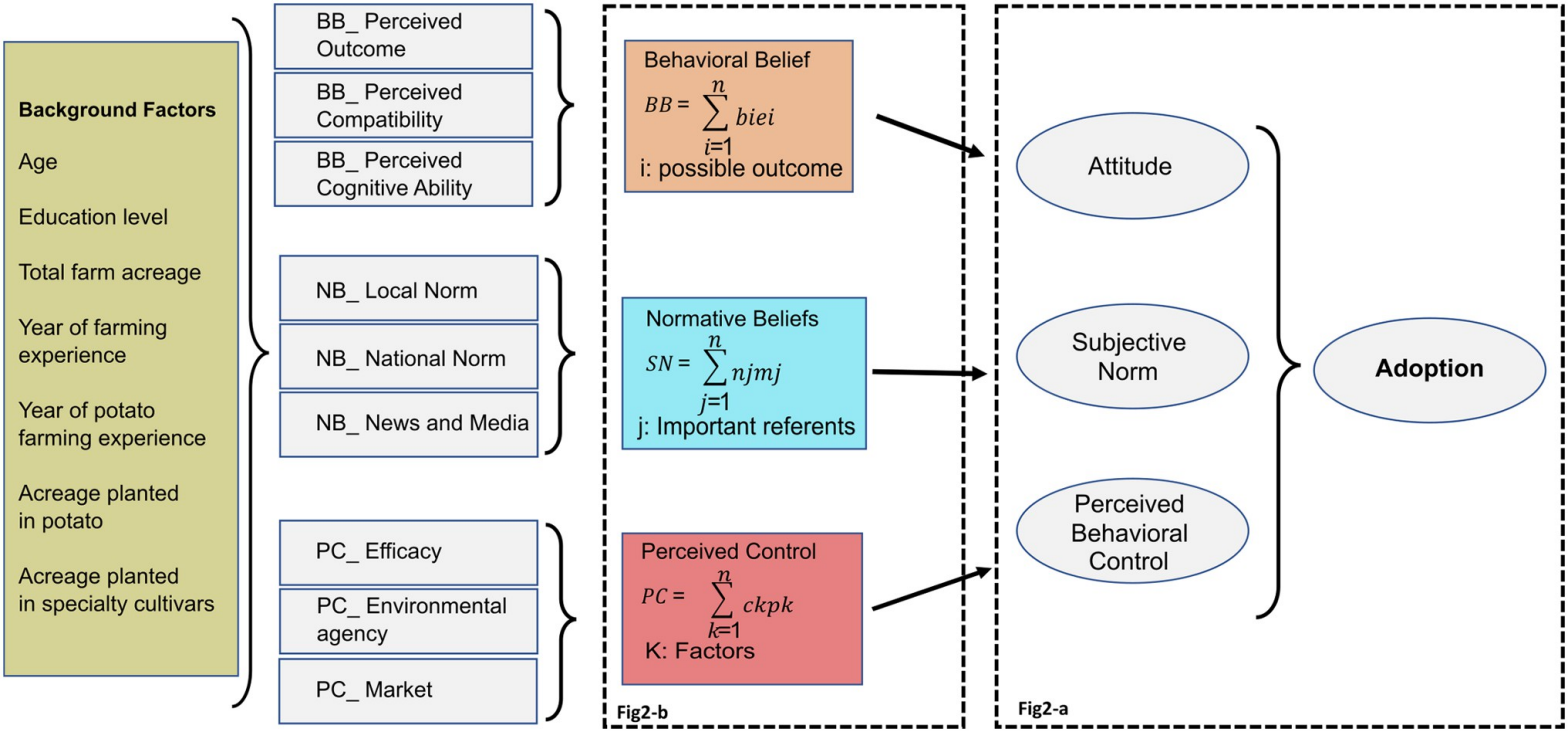
Theoretical framework for adoption of potato SCs. Background factors were collected for analysis of adoption in potato to model behavioral belief (BB), normative belief (NB), and perceived control (PC). (a): Three constructs of the Theory of Planned Behavior (TPB) model, adapted from Ajzen [37]. (b) Adaptation of the models of Borges et al. (2016) and Wauters et al. (2010) shows the aggregative effect of behavioral belief, normative belief, and perceived control on attitude, subjective norm, and perceived behavioral control [50, 76].

Based on the reviewed model components, agricultural organizations are not solitary decision-making entities whose decision-making processes can be predicted by individual socio-psychology factors. Social and economic factors simultaneously explain an agriculture organization’s decisions and adoption status [67, 70–72]. Changing behavior and shifting a grower’s decision to adopt a new SC result from multiple motivations and drivers. We construct an integrative theoretical framework to address the objective of our study (see Fig 2). We use Ajzen’s TPB components of behavioral belief, normative belief, and perceived control [37, 73] to explore grower attitudes and intention to adopt. Acknowledging that other factors may contribute to adoption, and as TPB is “in principle” open to inclusion of other predictors to capture other components that affect adoption, we integrate social and demographic factors with behavioral components [73–75].

### Study area and procedure

We conducted the study in the SLV, in south-central Colorado, which represents the third largest potato growing region in the US. It is a high alpine valley with sandy soils that is part of the Rio Grande Rift and stretches approximately 122 miles by 74 miles, surrounded by the San Juan Mountains to the west and the Sangre de Cristo Mountains to the east. Farming and ranching are the main sources of income in the San Luis Valley, and potatoes are the primary commodity produced [77].

**Fig 3:**
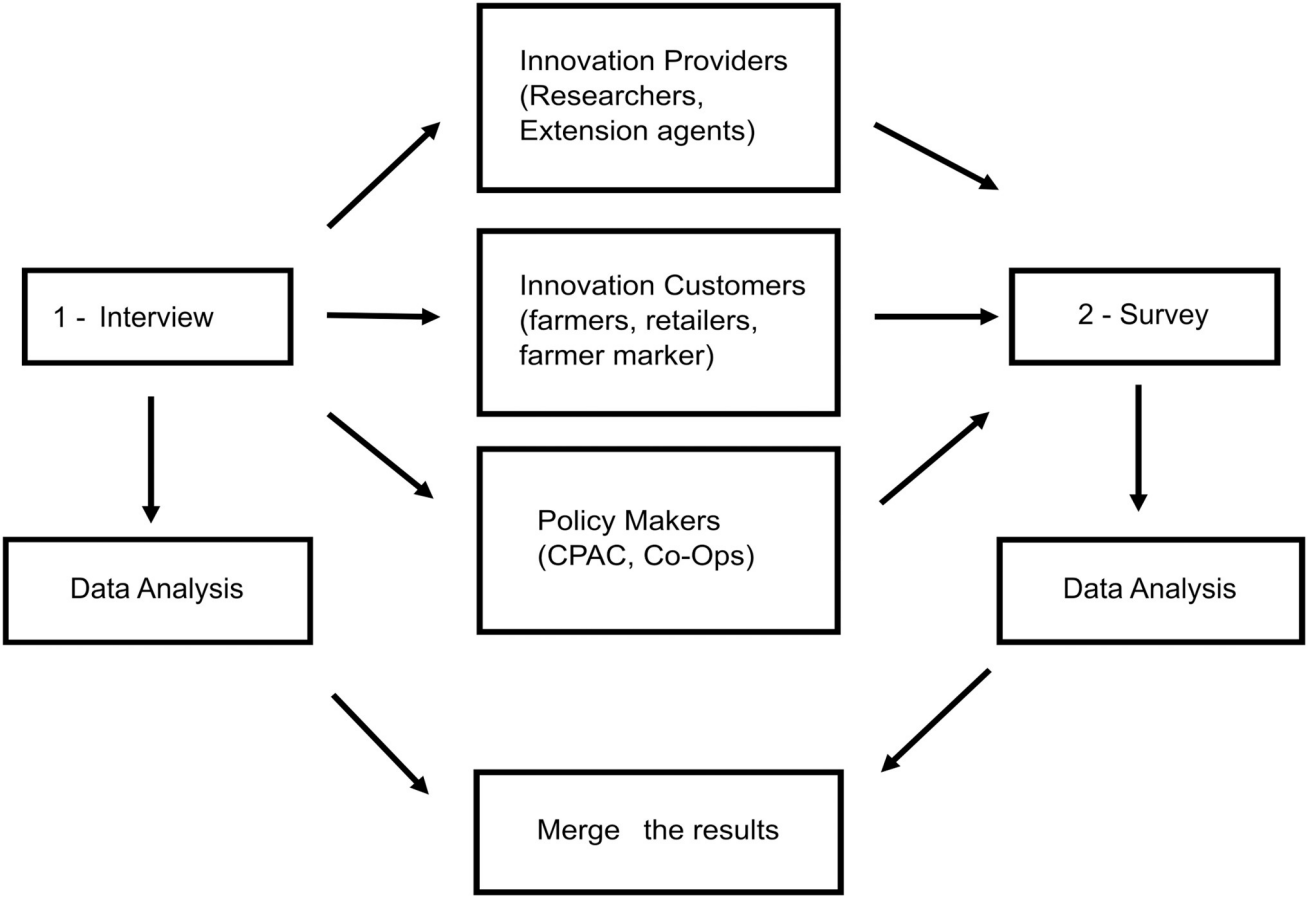
Experimental design to collect and analyze qualitative and quantitative data. The first step in the procedure was interviews, which we analyzed and used to generate the survey as the second step. Data from both the interview and the survey were merged for subsequent modeling.

We conducted the study in two steps (see Fig 3). First, we conducted interviews with participants within the potato growing community to contextualize the components of our theoretical model based on the conditions of potato growers in the SLV. Second, we conducted a survey to investigate the decision-making factors identified via interviews and a literature review. Note that as the survey did not include any identifying information about the respondent, it is possible that some potato growers participated in both the interview and survey. The survey included questions to model the following factors: (A) background (farm characteristics and grower demographic), (B) behavioral beliefs (outcome, compatibility, and cognitive), (C) normative beliefs (local, national news and media), and (D) perceived control (efficacy, environment, market).

#### Interviews

We conducted interviews to understand potential economic and social constraints and opportunities relative to adoption. We selected three groups for preliminary interviews: 1) Innovation Users (growers, household food users, retailers), 2) Innovation Providers (researchers, scientists, extension agents), and iii) Policymakers (local commodity groups, cooperatives). We initially characterized interviewees based on five categories/groups of innovators to match the Rogers innovation graph (Fig 1). We conducted semi-structured, face-to-face interviews with 20 growers. The 20 growers were selected to cover Rogers’s four adopter categories and identified through consultation with the Colorado Potato Administrative Committee (CPAC), members of Colorado State University’s (CSU) Potato Breeding and Selection Program, and regionally-based CSU extension agents. Interviews were recorded and later transcribed and analyzed. Questions were open-ended and descriptive. We used these interviews to capture the important outcomes (i), referents (j), and factors (k) involved in a grower’s intention to adopt, using Borges’ method [68, 76].

#### Survey

We developed a descriptive survey (open-ended, Likert scale, single answer, multiple-choice questions) at CSU, which is translated into descriptors and variable names in Table 1. The survey was initially tested on five growers and then reviewed by the Executive Director of the CPAC for content validity. We administered the survey in February 2019, with 80 potato growers responding (59%, out of the 135 total SLV potato growers indicated by CPAC); 76 of the responses were complete and used in the analysis. We administered the survey in person on tablet computers using Qualtrics [78] at an agricultural conference in the SLV. The survey included four sections to investigate our hypothesis: (A) background factors, (B) behavioral belief factors, (C) normative belief factors, and (D) perceived control factors. In the survey, we defined SCs as any potato other than white Russet potatoes (e.g., yellow, red, purple, fingerling, etc.). For translation of the survey to modeling, an adopter is defined as any grower who produced SCs in the previous growing season.

**Table 1:** Survey translated to descriptions and corresponding independent variables.

Variable class	Variable name	Description
Background factors	Adopter	Planted specialty potatoes in 2018 (1 if yes, 0 if no)
	Specialty Prct	Percent of potato acreage planted in non-Russet cultivar
	Year Farming	Years of total farming experience
	Year Potato	Years of potato farming experience
	T_Acres	Total farm acreage
	P_Acres	Total farm acreage planted in potatoes
	PrctAcresPotato	Acreage planted in potatoes divided by total acreage (P_Acres/T_Acres)
	Education	Highest level of education that grower completed (Less than high school = 1, High school = 2, Some college = 3, 4-year degree = 4, Graduate = 5)
	Age	As of December 31, 2018, what is grower age? (Years old)
Market channels	MC_W	Warehouses (1 if yes, 0 if no)
	MC_Chip	Chippers (1 if yes, 0 if no)
	MC_De	Dehydrators (1 if yes, 0 if no)
	MC_Ret	Retailers (1 if yes, 0 if no)
	MC_Expo	Export markets (1 if yes, 0 if no)
	MC_FM	Farmers markets (1 if yes, 0 if no)
	MC_etc	Other (e.g., bulk) (1 if yes, 0 if no)
	MC_Industry	Chippers, Dehydrators, Export (1 if yes, 0 if no)
	MC_FarmerNitch	Farmers markets or export markets, local consumers, retailers (1 if yes, 0 if no)
	MarketDiversity	Total number of market channels used by farm
Behavioral belief (BB) perceived outcome (i)	Per_HighYield	Higher yields (True = 3, Neither = 2, False = 1)
	Per_LowerCost	Lower cost of production (True = 3, Neither = 2, False = 1)
	Per_MoreProfit	More profitable (True = 3, Neither = 2, False = 1)
	Per_MoreMarket	More marketable (or subject to higher levels of competition) (True = 3, Neither = 2, False = 1)
	Per_BetterTaste	Taste better (True = 3, Neither = 2, False = 1)
	Per_Healthier	Healthier (True = 3, Neither = 2, False = 1)
BB perceived compatibility (ii)	Less Disease	Less prone to pest and disease pressure (True = 3, Neither = 2, False = 1)
	Per_LessLabor	Same labor requirements (True = 3, Neither = 2, False = 1)
	Per_NotDifficultStor	Less difficult to store (True = 3, Neither = 2, False = 1)
	Per_Certified	Lack certified standards (True = 3, Neither = 2, False = 1)
	Per_KnownConsumer	Less known/appreciated by consumers (True = 3, Neither = 2, False = 1)
BB perceived cognitive (iii)	Ati_Market	Over the next five years, the market for Colorado’s specialty potatoes will increase (Strongly agree = 4, somewhat agree = 3, somewhat disagree = 2, strongly disagree = 1, Neither = 0)
	Ati_Demand	There is a growing demand for specialty potatoes. (Strongly agree = 4, somewhat agree = 3, somewhat disagree = 2, strongly disagree = 1, Neither = 0)
	Ati_Health	Consumers are more concerned about the health benefits of the crop (Strongly agree = 4, somewhat agree = 3, somewhat disagree = 2, strongly disagree = 1, Neither = 0)
Normative beliefs (NB) local norms (i)	T_Neib Farm	Neighbor growers (extremely likely = 4, somewhat likely = 3, somewhat unlikely = 2, extremely unlikely = 1, neither = 0)
	T_CSU Ext	CSU Extension (extremely likely = 4, somewhat likely = 3, somewhat unlikely = 2, extremely unlikely = 1, neither = 0)
	T_SLVResearch	SLV research center (extremely likely = 4, somewhat likely = 3, somewhat unlikely = 2, extremely unlikely = 1, neither = 0)
	T_Industry	Commodity or industry organization (extremely likely = 4, somewhat likely = 3, somewhat unlikely = 2, extremely unlikely = 1, neither = 0)
NB national (ii)	T_P USA	National websites (extremely likely = 4, somewhat likely = 3, somewhat unlikely = 2, extremely unlikely = 1, neither = 0)
	T_USOrgNews	National websites, CDA, USDA (extremely likely = 4, somewhat likely = 3, somewhat unlikely = 2, extremely unlikely = 1, neither = 0)
NB news and media (iii)	T_S Media	Social media (extremely likely = 4, somewhat likely = 3, somewhat unlikely = 2, extremely unlikely = 1, neither = 0)
	T_News	Other websites and new outlets (extremely likely = 4, somewhat likely = 3, somewhat unlikely = 2, extremely unlikely = 1, neither = 0)
Perceived control (PC) efficacy (i)	Influ_EasePro	Ease of production (including all aspects from planting to harvest) (most influential = 8, least influential = 1)
	Influ_Maint	Maintains character in storage (most influential = 8, least influential = 1)
	Influ_etc	Other (Disease resistant, Seed availability, Marketability, Water use efficiency, etc.) (most influential = 8, least influential = 1)
PC environmental agency (ii)	Influ_News	Information or news from the university or commodity organization (most influential = 8, least influential = 1)
	Influ_Like	What grower family likes to eat or thinks tastes good (most influential = 8, least influential = 1)
	Influ_otherFarm	What other fellow growers like to plant (most influential = 8, least influential = 1)
PC market (iii)	Influ_GrewPrev	What farm grew or sold in previous year (most influential = 8, least influential = 1)
	Influ_GrewNeigh	What neighbor farm grows (most influential = 8, least influential = 1)
	Influ_Req	Request from a retailer or specific market (most influential = 8, least influential = 1)

*Perceived control (PC)*. We determined PC based on grower answers to questions where they were asked to rank the factors affecting their decision-making. We investigated the influence of different factors defined in the interviews to form data reported in Table 2 in three categories. First, we asked about Perceived Efficacy and ranked the influence of what their farm grew and sold last year, how easy and compatible it would be to use new cultivars, and the characteristics of new varieties when it came to maintenance and storage. Second, we designed Perceived Environment questions to assess the factors including neighboring farm behavior (e.g., if they grew SCs, if they received any specific demand from special consumers or commodities, and if their family liked to eat a specific cultivar). Third, we asked questions about growers’ perceptions of their control over the market to indicate the influence of their own or their neighbor’s production and sale in the previous year or season. Perception of control was also indicated as a factor of direct requests that they receive from various market sources.

**Table 2:** Perceived outcomes (i), important referents(j), and control factors(k) identified in semi-structured interviews.

Outcomes (i) to measure behavioral beliefs (b_i_e_i_)	Important referents (j) to measure normative beliefs (n_j_m_j_)	Factors (k) to measure control beliefs (c_k_p_k_)
Production: disease resistance, water usage, etc. Efficiency of resource and input Efficacy of storage and packaging Potential to sell to different market channels Social and professional prestige	Neighbor farms Retailors, warehouses, and industry Research and universities Local agriculture departments Media and news	Having a diversified operation Owning a sufficient operation Having access to different markets for sales Knowledge regarding new products Knowledge of sales and profits from previous years

### Empirical and statistical analysis

In this study, we defined the dependent variable (adoption status, binary as yes/no) based on whether a grower planted any SCs in their farm in the previous farming year (2018). Additionally, we calculated the level of adoption as the percentage of SC acres planted to total acres planted.

We ran a test of normality and analyzed the data using two different statistical platforms (GraphPad Prism, GraphPad Software, San Diego, California USA, and JMP Version 15, SAS Institute Inc., Cary, NC, 1989–2019) in two segments: descriptive statistics and inferential statistics. We calculated the mean, frequency, standard deviation (SD), and percentage for all growers in both the adopter and non-adopter groups. For inferential statistics, we first used Student’s t-test, chi-square, or Kruskal-Wallis to determine whether any of the differences between adopters and non-adopters are statistically significant (significance level of 0.05). Next, we calculated Spearman and Pearson correlation coefficients to assess the linear associations between independent variables and the dependent variable (adopters vs. non-adopters). The binary nature of adoption (yes/no) allows us to model the probability of adoption, depending on the other variables, in randomly selected subsamples from the population. We utilized a logistic binary choice model to provide a method for modeling adoption as a binary response variable, which takes values 1 (for adopters) and 0 (for adopters), to estimate this probability and predict the effect of a variable on the responses. We used a logistic algorithm because it does not require linearity between dependent and independent variables and does not assume homoscedasticity. We used Wald χ2 statistics to test the significance of variable coefficients in the model, comparing each Wald statistic with an χ2 distribution with 1 degree of freedom. We merged the qualitative and quantitative data in the analysis and interpretation phases of the study. This model predicted the probability of the response level (1 for adopter or 0 for non-adopter), given the value of the independent variables (socioeconomic factors, perceived attitude, norms, and controls) [79, 80]. We defined our logistic model as the equation:

$$In[Pi/\;((1-P_{i})]=\beta 0\;+\;\beta 1X1_{i}\;+\;\beta 2X2i\;+\;\ldots \ldots \ldots +\;\beta kXki$$

where the subscript i is the i^th^ grower in the sample. P is the probability that a grower adopts the SCs and (1-P) is the probability that a grower does not adopt the SCs. The regression of P on X_i_s estimates the parameter of βk (1,2, …k) via the maximum likelihood method. In logistic regression, we tested the probability that all βs = 0 versus at least one β is not zero, and we determined probabilities using the chi-square likelihood ratio test. The βs are considered a regression coefficient and indicate the effect of a one-unit change in the variable X_i_ on the log of the odds when the other variables are held constant. The distribution probability of the difference between the full model (containing all βs) and the reduced model (nested model) is calculated by the -Log-Likelihood value. Therefore, we used the chi-square test to assess how independent variables improved the model fit (Table 1). The chi-square probability of the difference between these models explains whether the model is reliable or not. The improvement in model fit with each added predictor is shown by statistical significance between the two models (Petrucci, 2009). The resulting classification matrix is a summary and can be used to calculate the specificity and sensitivity of the model. From the classification matrix, we calculated the percentage of the growers who are adopters and correctly identified by our model (the sensitivity of the model).

### Consent and ethics statement

This study was approved by Revised Human Subjects Regulations (Colorado State U IRB #19-8659H). The respondents were guaranteed confidentiality and given the right to decline to answer any question to which they were not comfortable responding. All participants gave written informed consent before participating in the study.

## Results

### Interview and identification of theory of planned behavior components

From the interviews, we extracted five major groups for each component of the TPB (Table 2). The outcomes that were important for the growers and would affect their decision to plant SCs were: (1) if the production of a new cultivar (seeding to harvest) would be different from that of conventional cultivars (2) if they would need a different storage and packaging system to plant new cultivars, (3) if their resources (financial and operational) could handle the variation in production of these new cultivars, (4) if these new cultivars have markets, and (5) if planting new cultivars brings prestige and respect to their farm.

Growers reported their social networks and their relationships with local and national institutional stakeholders (i.e., CPAC, Potato USA). Growers were then asked whose opinion within their social and policymaker networks positively or negatively affects their decisions. Accordingly, we identified five groups based on important references (j) as components of the network with an influence on adoption (Table 2). Table 2 shows five factors (k) that growers reported as facilitating or inhibiting their control in producing SCs. These factors include: (1) having a diverse operation that produces more than one product (i.e., potato seed, other crops), (2) owning sufficient infrastructure (i.e., storage and packing capacity), (3) having access to markets demanding SCs (i.e., restaurants), (4) receiving a direct request from specific buyers or markets, and (5) having sufficient knowledge of the benefits of new cultivars. We then used these i, j, and k data acquired from the initial interviews to build the survey to explore SC adoption in more detail.

### Survey respondent socioeconomic characteristics and associations with adoption

The response rate of the survey indicated broad participation within the SLV. Characteristics of the respondents and the farms are shown in Table 3. Based on our respondents, approximately 74% of the potato farmland area in the SLV was dedicated to Russet (the conventional market class), while 26% was allocated to growing SCs. Of those who planted SCs, 43% (SD 34%) of their land area was devoted to SCs. A total of 46 of the 76 respondents reported planting some SCs in their operation in 2018 and were therefore classified as adopters (61%).

**Table 3:** Farm and grower characteristics.

Characteristics		Obs	Mean	SD	Min	Max	Prob
Total farm size (hectares)	All	76	597	314	51	1214	
	Adopter	46	682	328	51	1214	
	Non-adopter	30	464	239	129	963	
	Adopter vs. non-adopter						0.01
Land dedicated to potatoes (hectares)	All	76	301	231	39	991	
	Adopter	46	383	226	45	991	
	Non-adopter	30	175	106	39	505	
	Adopter vs. non-adopter						< 0.01
Farming experience (years)	All	76	28	15	3	70	
	Adopter	46	21	11	3	50	
	Non-adopter	30	38	15	10	70	
	Adopter vs. non-adopter						<0.01
Potato growing experience (years)	All	76	23	16	1	70	
	Adopter	46	18	11	2	40	
	Non-adopter	30	30	13	1	70	
	Adopter vs. non-adopter						0.03
Education level (Likert scale 1–5)	All	74	3.67	0.90	2	5	
	Adopter	44	3.95	0.86	2	5	
	Non-adopter	30	3.26	0.82	2	5	
	Adopter vs. non-adopter						0.01
Age (years)	All	76	48	15	20	84	
	Adopter	46	42	13	20	65	
	Non-adopter	30	56	14	29	84	
	Adopter vs. non-adopter						< 0.01

Adopters were significantly younger on average than non-adopters (42 compared to 56, respectively) (Kruskal-Wallis adjusted P < 0.01). Approximately 93% of all respondents have education above a high school diploma, with 23% having a college degree, 42% having a 4-year college degree, and 19% having a graduate degree. Education level also differed between adopters and non-adopters, with adopters having higher education levels (Kruskal-Wallis adjusted P < 0.01).

Farming experience varied widely for all respondents (3 to 70 years, mean = 28, SD = 15), including experience farming potatoes (1 to 70 years, mean = 24, SD = 17). Non-adopters had both more farming experience overall (39 years compared to 21 years) and more experience farming potatoes (30 years compared to 19 years). The mean farm size of respondents was 597 hectares (SD = 314, converted from acres reported in the survey), and approximately half of the farm was dedicated to potatoes each growing season (mean of 301, SD = 231). Adopters had significantly greater hectares cultivated (Kruskal-Wallis adjusted P = 0.10) and a greater share of their land dedicated to potatoes (57% in adopters vs. 42% in non-adopters with Kruskal-Wallis adjusted P < 0.01).

### Components of growers’ attitudes, subjective norms, and perceived control toward planting specialty cultivars

The behavioral belief was comprised of the three subgroups: perceived outcome, perceived compatibility, and perceived cognitive ability (Fig 2). The major themes that emerged from the survey were the belief that SCs have better outcomes for operations, such as marketability and profitability (Table 4, mean = 1.82), though adopters had higher positive perceived outcomes towards SCs compared to non-adopters (Kruskal-Wallis adjusted P < 0.01m, Table 4). In the outcome questions, adopters and non-adopters demonstrated similar attitudes about taste and health attributes unique to SCs (Chi Square = 0.06 and 0.91). However, adopters believed that SCs are more marketable (Chi Square Likelihood ratio < 0.01) and can bring more profit to their operations (Chi Square Likelihood ratio = 0.01).

**Table 4:** Distribution of adopters and non-adopters by behavioral components.

Variable	Group	Obs	Mean	SD	Min	Max	Prob^b^
PC_Personal efficiency^a^	All	76	5.04	0.52	3.75	6.50	
	Adopter	46	4.90	0.51	3.75	6.00	
	Non-adopter	30	5.25	0.48	4.00	6.50	
	Adopter vs. non-adopter						0.01
PC_Environmental agency	All	76	3.13	0.59	2.00	5.00	
	Adopter	46	3.23	0.62	2.00	5.00	
	Non-adopter	30	2.97	0.53	2.00	4.33	
	Adopter vs. non-adopter						0.14
PC_Market	All	76	5.03	1.05	2.00	7.00	
	Adopter	46	5.29	1.07	2.00	7.00	
	Non-adopter	30	4.63	0.89	2.00	6.33	
	Adopter vs. non-adopter						< 0.01
SN_Local norm Local norm	All	75	2.37	0.81	0.00	4.00	
	Adopter	45	2.64	0.60	1.25	3.75	
	Non-adopter	30	1.97	0.91	0.00	4.00	
	Adopter vs. non-adopter						< 0.01
SN_National norm	All	74	1.58	1.13	0.00	4.00	
	Adopter	44	1.67	1.07	0.00	3.50	
	Non-adopter	30	1.46	1.23	0.00	4.00	
	Adopter vs. non-adopter						0.03
SN_ Media	All	71	1.27	1.03	0.00	4.00	
	Adopter	43	1.28	0.90	0.00	3.00	
	Non-adopter	28	1.26	1.22	0.00	4.00	
	Adopter vs. non-adopter						0.83
BB_Perceived outcome	All	72	1.82	0.36	1.00	2.66	
	Adopter	43	1.98	0.34	1.16	2.66	
	Non-adopter	29	1.59	0.26	1.00	2.16	
	Adopter vs. non-adopter						< 0.01
BB_ Perceived compatibility	All	73	1.70	0.38	1.00	3.00	
	Adopter	43	1.70	0.35	1.20	3.00	
	Non-adopter	30	1.71	0.42	1.00	2.80	
	Adopter vs. non-adopter						0.99
BB_ Perceived cognitive	All	74	3.07	0.83	1.00	4.00	
	Adopter	44	3.31	0.62	1.00	4.00	
	Non-adopter	30	2.72	0.96	1.00	4.00	
	Adopter vs. non-adopter						< 0.01

^a^PC: Perceived Control; SN: Social Norm; BB: Behavioral Belief

^b^ Chi Square Likelihood Test for adopters vs. non-adopters

All respondents (adopters and non-adopters) believed that SCs are compatible with their traditional farming operations and equipment. Growers noted that the SCs are not more susceptible to disease, nor did they need any special labor requirements. Both groups believed that these cultivars are not well known by consumers (41% of non-adopters and 58% of adopters with Chi Square = 0.24).

We evaluated growers’ perceived cognitive ability by asking if they think there is an increasing demand for healthy and more nutritionally valuable crops. We also asked if they think SCs have a higher health benefit compared to conventional varieties. Finally, we asked if enhanced health attributes would lead to a larger market in the next 5 years. Both groups were unsure if the market for SCs would increase in the next five years, largely due to current certification problems (e.g., federal and state inspections) and low consumer awareness of their benefits (47% of non-adopters and 52% of adopters with Chi Square = 0.06). Nevertheless, adopters had a significantly higher positive perceived cognitive ability (Kruskal-Wallis adjusted P < 0.01).

For subjective norms, questions in three subgroups qualified the reliance of the grower on their important references and information sources. We asked growers to rank how likely they are to rely on different sources of information. Trust in the local agricultural advisory groups and local peer growers was higher in adopters (Kruskal-Wallis adjusted P < 0.01). Factors of social media and national agricultural websites did not influence grower decisions, though the adopters trended towards a higher reliance on national agricultural websites vs. non-adopters.

We asked growers about their perceived control over the cultivation of and experience with the market for SCs (perceived efficacy). Control of the production process was an important factor for all growers when deciding what to plant. Adopters had higher positive perceptions of their personal efficacy for planting SCs (Kruskal-Wallis adjusted P = 0.01). The planting decisions of both the adopter and non-adopter groups were affected by what neighboring farms could sell the year before and requests from industry or retailers. Further, adopters had a more positive perception of the market for SCs (Kruskal-Wallis adjusted P < 0.01). The difference between subcategories means for control efficacy of the market were not significant. Further, retailers, chippers and dehydrators, and farmers’ markets are the markets to which growers have access in the SLV. Importantly, the data indicate that adopters utilized several of these markets, while non-adopters only sold their products to warehouses.

### Modeling the adoption status and decision-making process of growers

The regression results of the Logit model include the coefficients (β), their standard errors, the Wald Chi Square statistic, connected P, and marginal probability (marginal effects), all of which are reported in Table 5. We tested the statistical significance of individual regression coefficients (βs) using the Wald Chi Square statistic reported in Table 4. The model demonstrates that several variables positively influence adoption behavior, including local norms (β = 2.87 and P < 0.01), market diversity (β = 1.16 and P < 0.01), BB_Perceived Outcom (β = 1.77, P < 0.01), PC_Perceived Market (β = 2.69, P < 0.02), and BB_Perceived Cognitive (β = 14.74, p < 0.01). Years of farming experience, and specifically potato farming experience, trended towards having a negative influence on adoption behavior (β = -0.10, P < 0.06).

**Table 5:** Factors influencing the adoption of SCs determined by logistic regression*.

Variable	β	Std Error	Wald Prob>ChiSq	Prob>ChiSq
BB_Perceived Outcome	1.77	4.95	< 0.01	0.03
SN_Local	2.87	1.21	0.01	0.01
BB_Perceived Cognitive	14.74	1.10	0.01	0.01
PC_Percieived Market	2.69	0.77	0.02	0.02
Market Diversity	1.16	0.55	0.03	0.03
Year Potato	-0.10	0.05	0.06	0.06
Intercept	-50.26	16.65		

*PC: Perceived Control; SN: Social Norm; BB: Behavioral Belief

We evaluated the whole model and found it to be reliable (Table 6, -Log likelihood < 0.0001, R^2^ = 0.70). We also evaluated the model’s accuracy via a classification matrix (Table 7). The matrix shows that the model can correctly classify a grower as an adopter with 90% precision and can identify non-adopters with 82% sensitivity. Thus, the overall model is significant, and the variables used in the model are together able to explain grower behavior regarding the decision to adopt potato specialty varieties:

$$\begin{array}{c} log\left(\frac{p}{1}-p\right)=-50.26\;+\;1.16\;Market\;diversity\;+\;2.87\;{SN}_{Local}+\;1.77\;{BB}_{Perceived}Outcome\\ +14.47\;{BB}_{Perceived}Cognitive+2.69\;{PC}_{Percieived}Market-0.10\;Year\;Potato \end{array}$$

**Table 6:** Whole model test.

AUC	R^2^	- Log likelihood (Prob>ChiSq)	Misclassification rate
0.975	0.70	<0.0001	0.125

**Table 7:** Classification matrix to compare the actual vs. predicted outcome.

	Actual	Predicted
Adopter	1	0
1	39	4
0	5	24
Precision	90%	
Sensitivity	82%	

## Discussion

This article presents an integrated theoretical framework that identifies factors influencing the decision of potato growers in the SLV to adopt SCs. We leverage the Diffusion of Innovation Model, Rational Expectations Hypothesis, and Theory of Planned Behavior, as we found these have not previously been used collectively to analyze and predict grower adoption of new SCs as a combined model. In this case study of potatoes, a grower’s decision to adopt was primarily shaped by their attitude, subjective norms, and perceived behavioral control, combined with some background factors (Fig 2).

Our results show that access to different market channels and having a secure mechanism for sales is the primary driver to adopting SCs. Access to diverse market channels (e.g., farmers’ markets, restaurants) appears to give potato growers a higher perception of the profitability benefits associated with SCs. Adopters, on average, also seek more information to help them diversify their markets, while non-adopters tend to rely on fewer, larger-scale buyers (i.e., warehouses). Consequently, adopters appear more likely to seek out information from local sources of educational information including the local agricultural research center and breeding program.

### Some components of attitude were related to the adoption of specialty cultivars

Previous research has identified that yield and disease resistance are important factors in adopting new cultivars [54]. This study found that growers’ attitudes were shaped by their assessment of compatibility, outcomes, and their cognition. However, both adopters and non-adopters agreed that SCs are compatible with their operating systems. Further, though SCs are usually smaller in size and sometimes more sensitive to manage, the growers in the study, on average, felt that they have enough experience to address any required modifications to the cultivation of SCs.

On average, growers were also aware of unique SC traits of distinct flavor/color, cooking properties, and human health benefits. Both adopters and nonadopters agreed that SCs have novel traits, but that there is a significant lack of knowledge and awareness among consumers and the industry that impacts the marketability of these cultivars. This perceived awareness gap has resulted in a lack of certification and marketing for the new cultivars, which further increases the risk of not being accepted within the market.

Attitude differences between the two groups were evident in perceived cognitive abilities and outcomes. Adopters noted that the market for potatoes is changing, and consumers are looking for specialized products. Adopters also believed that the SC traits of smaller tuber size and larger variation in color will be attractive to consumers, although they stated that these cultivars have not been advertised and introduced to the market properly (this information was derived from the interviews and not included in the model). In addition to the physical attributes of SCs, adopters believed the higher health benefits of SCs can be a significant factor in sales and may lead to greater profits, which is consistent with other studies that demonstrate consumer value of and willingness to pay for healthier products. For example, a study in Ohio (USA) found that consumers were willing to pay more for scientifically backed claims of healthier tomato juice [81]. In Europe, similar trends were observed for cardioprotective foods in Germany [82] and in willingness to pay more for healthier dairy products [53, 83]. This increased willingness to pay for healthier food products is also demonstrated in staple plant foods such as rice (Cuevas et al., 2016), potatoes [84], and healthier potato chips [85]. Altogether, these studies support that consumer knowledge and awareness of the health benefits of SCs can play an important role in increasing grower adoption of new cultivars, consumer access to SCs, and, ultimately, grower profitability.

### The relationship between background factors and attitude

We observed that farming experience has a positive correlation with growers’ perceived compatibility, but a negative correlation with their perceived outcomes. The positive correlation between years of farming experience (specifically potato farming) and perceived compatibility may mean that experience enables a positive attitude towards the management and cultivation of new SCs. This finding is consistent with that reported by Danso-Abbeam (2017) and Ojo (2014): adoption of new varieties of maize is higher among growers with more years of experience in agricultural production [86, 87]. Other research has similarly found that years of experience is positively related to the adoption of new technologies [41, 88–90]. However, as opposed to some previous research, we find that farming experience is negatively related to producers’ perceived outcomes associated with SC adoption. Based on the interviews, we believe this is due to the perception of more experienced growers that they know and understand the market. After years of farming, growers develop strong relationships with buyers (e.g., warehouses) and have less need and desire to take on more risky marketing relationships.

### Subjective norms and modeling adoption

This research finds that local networks are an important factor in influencing the adoption of SCs. This is consistent with previous research that finds, for example, that growers who participate in trainings and workshops offered by university and extension services have a higher probability of adopting an innovation [54, 86, 91, 92]. Similarly, local grower-based organizations and networks have been also identified as factors that affect a grower’s attitude towards innovation [68, 91, 93]. In general, grower-to-grower networking is as important as a grower’s SC experience (Fig 1, early stage experience as “innovators” and “early adopters”), as it can give peers a different perspective on a new situation. The influence of a peer grower’s experience can improve a grower’s perception of the outcome. This is aligned with other research that shows how a grower’s experience with a new technology or cultivar positively affects the overall adoption within a network [65, 94–96]. A grower’s positive experience with either production or the sale of a new crop in a previous year increase the availability of information and skills for others and increases the probability of adoption [97]. Accordingly, should the adoption of SCs be found profitable for adopters, our results point to the importance of university extension programs working with growers on training, as well as facilitating grower-to-grower learning opportunities.

While both the adopter and non-adopter groups did not rely on news from national agricultural websites, they relied heavily on social media (more significantly within the adopter group). This may be attributed to the premise that adopters use social media to connect with their consumers and obtain feedback from niche markets. Social media may also be a source of information for adopters to understand changing consumer interests and behaviors.

Interestingly, growers that were older and more experienced were less likely to be adopters. Previous research has also found that age is a barrier to adopting new technology or a new product [54, 86], but there is little understanding of why age matters. In our study, we found that age correlated with a grower’s trust in norms (local and national) and belief in the importance of avoiding risk. Perhaps our results are due to the fact that younger and less experienced farmers (adopters) tended to gather information through more different sources when evaluating a new market compared to older growers.

### Perceived behavioral control: An interconnection to norm and attitude

A grower’s perception of their ability to operate and manage the production and sale of a new crop on their farm has been analyzed in several models as a predictor of adoption (e.g., [98]). In this study, we observed that a grower’s perceived control varies, as adopters had higher perceived efficacy of the production to cultivate, harvest, store, and, most importantly, sell the SCs. This belief in control over the cultivation and sale also shows a correlation with growers who trust local norms. Most of the positive correlation with local resources was due to related university breeding programs, further supporting that a university and extension service can empower growers to diversify their operations.

The most important influence of a grower’s perceived control is their belief in personal control over the market. Both groups were attentive to requests from the market (e.g., warehouses, local restaurants, farmers markets). In this potato study, demand from local market for SC in previous year reduced the perceived risk for potato growers. Adopters used many more markets than non-adopters. Based on the interview responses, this likely reflects the fact that the primary larger-scale market (warehouses), largely did not purchase SCs. Accordingly, adopters had to diversify the markets they used, which in turn appeared to provide adopters with an added sense of control.

Interestingly, we also find that younger growers were more likely to use diverse channels. This may reflect the fact that warehouses have long-term relationships with growers, and those younger producers are less likely to have these relationships. Further, growers that access niche markets have the ability to more easily transmit information about SC traits to customers, which may reduce reliance on experience from past year’s sales. This contrasts with non-adopters, who tended to be heavily influenced by their neighbors’ experiences and were more deterred from adopting new SCs by prior poor sales and experiences.

## Conclusion

This study reveals the major barriers to and influencing factors for how growers decide to adopt SCs in SLV’s potato industry. University participatory breeding and extension is important to facilitate adoption of new cultivars and is sufficient in transmitting knowledge of specialty potatoes, their unique traits, and their potential markets. Potato growers have a strong awareness of new SCs and can manage them with little changes to regular farm operations. However, widespread awareness of the benefits of SC traits, such as flavor and human health, is lacking and represents a key step to broader adoption of SC especially by more established growers. Based on these data, the major recommendations for the potato industry include: i) increased dissemination of knowledge regarding unique traits to distributors and consumers; ii) continued and enhanced agricultural extension services with knowledge of new specialty cultivars; iii) improved certification and packaging for specialty cultivars; and iv) promotion and improved focus on the utility of specialty cultivars in the market–particularly those that are larger scale.

## Abbreviations

BB: Behavioral Belief
CPAC: Colorado Potato Administrative Committee Area II
DOI: Diffusion of Innovation
PC: Perceived Control
REH: Rational Expectations Hypothesis
SCs: Specialty Cultivars
SLV: San Luis Valley
SN: Subjective Norms
TPB: Theory of Planned Behavior
